# Painful nodules on the feet

**DOI:** 10.1016/j.jdcr.2025.11.040

**Published:** 2025-12-05

**Authors:** Celine Jessica Lee, Jennifer Nguyen, Senhong Lee, Francis Yi Xing Lai

**Affiliations:** a College of Medicine and Dentistry, James Cook University, Townsville, Queensland, Australia; b Dermatology Department, Monash Medical Centre, Clayton, Victoria, Australia; c School of Medicine, Monash University, Clayton, Victoria, Australia; d Dermatology Department, Eastern Health, Box Hill, Victoria, Australia

**Keywords:** complex regional pain syndrome, idiopathic plantar hidradenitis, neutrophilic eccrine hidradenitis, pediatric dermatology, reflex sympathetic dystrophy

A previously healthy and active 10-year-old girl presented during winter with painful nodules on the plantar surfaces of bilateral feet, causing significant difficulty in mobilization. She had no recent illness, joint pain, fevers, trauma, or other systemic symptoms. On examination, multiple swollen violaceous nodules were distributed in the arches of bilateral plantar feet (as depicted in Fig 1). There was no involvement of the palms, lymphadenopathy, or other cutaneous findings. Full blood count, electrolytes, renal and liver function, C-reactive protein, erythrocyte sedimentation, and vasculitis screen were normal.

**Fig 1:**
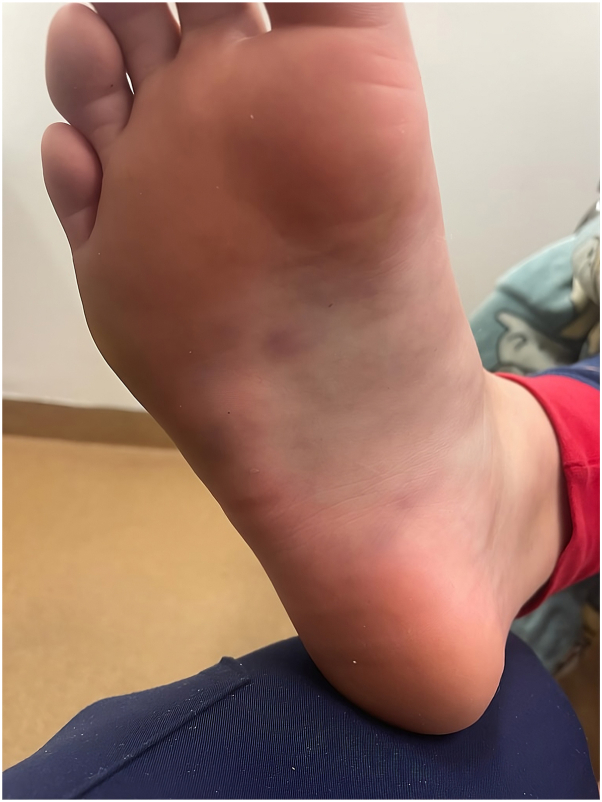
Image of the patient’s right plantar surface with multiple violaceous nodules.

Incisional biopsy, shown in Fig 2, demonstrated eccrine and apocrine sweat glands at the periphery of a suppurative abscess, fat necrosis, neutrophilic infiltration, and macrophages.

**Fig 2:**
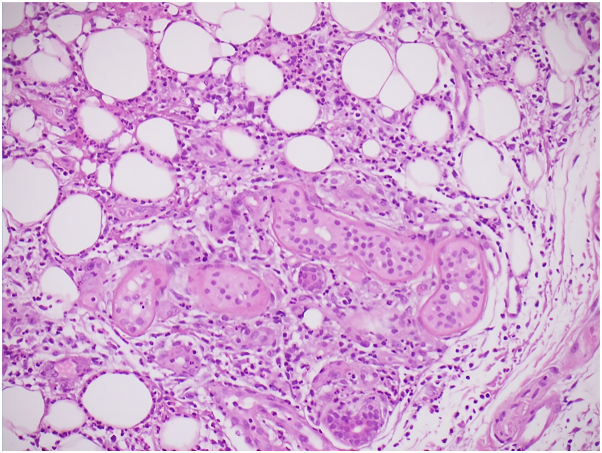
Histopathology demonstrating neutrophilic infiltration, and macrophages surrounding sweat glands (hematoxylin-eosin stain, magnification ×400).

**Question: What is the diagnosis?**

**A.** Idiopathic plantar hidradenitis
**B.** Delayed pressure urticaria
**C.** Erythema nodosum
**D.** Vasculitis
**E.** Chilblains

**Answer:**

**A.** Idiopathic plantar hidradenitis (IPH) is a rare, self-limiting dermatologic condition affecting children and young adults.^1^ IPH is characterized by the sudden onset of painful nodules on the soles or, less commonly, the palms.^1^ Risk factors include thermal and mechanical trauma, intense physical activity, exposure to moist environments, or excessive sweating.^2^ These factors are hypothesized to contribute to eccrine gland rupture, initiating an inflammatory response, leading to neutrophilic infiltration and inflammatory cytokine activation. Diagnosis is primarily clinical, although histopathology remains the gold standard.^2^

Treatments typically include conservative measures such as bed rest and analgesia. Some cases require topical and/or oral steroids, colchicine, or antibiotics for suspected infections.^3^^,^^4^ This patient was initially managed with bed rest, analgesia, and betamethasone dipropionate 0.05% ointment. Symptoms resolved within a month of treatment, however, recurred 2 weeks later with no obvious triggers, necessitating colchicine 500 micrograms 3 times a day and a 3-week weaning prednisone course. The nodules and pain gradually resolved after 3 months. However, 4 months following, while still on colchicine, she developed pain, without clinically obvious nodules, predominately in the right plantar foot. She had allodynia and hyperalgesia upon light touch. She had reduced foot range of motion, walked in a tip-toe manner, and used crutches. A magnetic resonance imaging foot ruled out pathology that would account for the patient’s symptoms.

Multiple treatments were used: prednisone 25 mg and 12.5 mg for 1 week each, ciprofloxacin 500 mg daily for 2 weeks, dapsone 50 mg daily, betamethasone dipropionate 0.05% ointment, paracetamol, ibuprofen, and topical amitriptyline 2%. These therapies were inadequate for her pain management and were eventually ceased. She was referred to the pain specialist and was subsequently diagnosed with complex regional pain syndrome as she fulfilled 3 of 4 of the Budapest Criteria, including clinically disproportional pain, sensory and motor signs and symptoms, and there was no other diagnosis that better explained her signs and symptoms.^5^ She underwent treatment with oral amitriptyline, gabapentin, and physiotherapy for desensitization therapy resulting in significant pain reduction. She returned to her pain-free baseline after 6 months.

While most cases of IPH respond well to conservative management, this case demonstrates a prolonged and recalcitrant course with negative sequelae of pain sensitization requiring multimodal treatment.

## Conflicts of interest

None disclosed.
